# Computation and Evaluation of Features of Surface Electromyogram to Identify the Force of Muscle Contraction and Muscle Fatigue

**DOI:** 10.1155/2014/197960

**Published:** 2014-06-04

**Authors:** Sridhar P. Arjunan, Dinesh K. Kumar, Ganesh Naik

**Affiliations:** ^1^Biosignals Lab, School of Electrical and Computer Engineering, RMIT University, GPO Box 2476, Melbourne, VIC 3001, Australia; ^2^Faculty of Engineering and Information Technology (FEIT), University of Technology Sydney (UTS), Sydney, NSW 2007, Australia

## Abstract

The relationship between force of muscle contraction and muscle fatigue with six different features of surface electromyogram (sEMG) was determined by conducting experiments on thirty-five volunteers. The participants performed isometric contractions at 50%, 75%, and 100% of their maximum voluntary contraction (MVC). Six features were considered in this study: *normalised spectral index (NSM5), median frequency, root mean square, waveform length, normalised root mean square (NRMS), and increase in synchronization (IIS) index*. Analysis of variance (ANOVA) and linear regression analysis were performed to determine the significance of the feature with respect to the three factors: muscle force, muscle fatigue, and subject. The results show that IIS index of sEMG had the highest correlation with muscle fatigue and the relationship was statistically significant (*P* < 0.01), while NSM5 associated best with level of muscle contraction (%MVC) (*P* < 0.01). Both of these features were not affected by the intersubject variations (*P* > 0.05).

## 1. Introduction

Surface electromyogram (sEMG) is the recording of the electrical activity that is associated with muscle activation [1]. sEMG is the interferential summation of tissue-filtered motor unit action potentials (MUAP) generated by active motor units and represents a pattern characterizing the general state of the muscle examined [2, 3].

Strength of muscle contraction is dependent on the number of active motor units, their size, the rate of stimulation of the motor units, and the type of muscle fibres. The ability of the muscle to contract and produce force can diminish over sustained contraction and when it is localized to a muscle or group of muscles that is referred to as localized muscle fatigue [4, 5] and is also closely associated with sEMG. Numerous studies [6–14] have reported the relationship of sEMG with force of muscle contraction and localized muscle fatigue.

Various features of sEMG such as root mean square (RMS) [6], median frequency [7], wavelet transforms [8, 9], fractal dimension [10, 11], normalized spectral moments [12, 13], and increase in synchronization (IIS) index [14] have been related to parameters of muscle contraction such as force and muscle fatigue. However, there are a number of compounding factors such as force of contraction, onset of muscle fatigue, length of the muscle, tissue properties, and external factors such as noise and intersubject variations that influence sEMG, and, thus, sEMG is considered to be suitable for only measuring the relative change in the muscle state [15–17].

Surface EMG is noninvasive and easy to record signal and machine based estimation of force of muscle contraction or for assessing muscle fatigue it will have large number of rehabilitation and other applications. However, while a number of studies have identified different features of sEMG and demonstrated the association of these with force and fatigue, no study has compared the relationship of these features and evaluated these features for automated estimation of force and fatigue from sEMG.

The aim of this study was to experimentally determine the most suitable of the currently used sEMG features that can be implemented for machine based sEMG analysis to estimate muscle force and fatigue. This research has experimentally studied six well-accepted features of sEMG and analyzed the relationship of each of these with force of muscle contraction and with muscle fatigue. Linear regression and analysis of variance (ANOVA) were performed to compare the relationship of each of these features with the force of muscle contraction and muscle fatigue. The significance of this study is that it has shown a comparison between the various features of sEMG that have been reported in literature and has identified the relationship of differences due to three factors:* subject, force, and fatigue*.

## 2. Materials and Methods

### 2.1. sEMG Recording

sEMG signals were recorded using the proprietary Delsys (Boston, MA, USA) sEMG acquisition system. The system supports bipolar recording and has gain of 1000, CMRR of 92 dB, and bandwidth of 20–450 Hz, with 12 dB/octave roll-off. The sampling rate was fixed at 1000 samples/second, and the resolution was 16 bits/sample. Active bipolar electrodes (Delsys, Boston, MA, USA), having two silver bars (1 mm wide and 10 mm long) mounted directly on the preamplifier with fixed interelectrode distance of 10 mm, were used for recording sEMG.

The experiments were conducted on the biceps brachii muscle because this muscle is superficial and is the prime mover for the ankle joint, and the muscle fibers run parallel to the surface. Two bipolar electrodes were placed above the motor points of the short head of the biceps brachii muscle and inline between the anticubital fossa (depression in the front of the elbow—lateral to the biceps brachii tendon) and the acromion process (part of the scapula which extends over the shoulder), at 1/3rd distance from the anticubital fossa. The distance between the two bipolar electrodes was maintained at 2 cm. Reference electrode was placed on the dorsal section and under the elbow. Prior to electrode placement, the skin area was cleaned with alcohol swabs and lightly exfoliated with paper towel to reduce skin impedance and ensure good adhesion of the electrodes.

### 2.2. Experimental Protocol

Thirty-five healthy subjects (22 male and 13 female, aged 22–35 years) consented to participate in these trials. The participants with the following criteria were excluded: (1) arthritis (e.g., osteoarthritis and rheumatoid arthritis); (2) neuromuscular disorders including collagen disorders and nonarticular rheumatism including fibro myalgia or seizure disorders; (3) any recent injuries of the hand, wrist, or arm.

**Figure 1:**
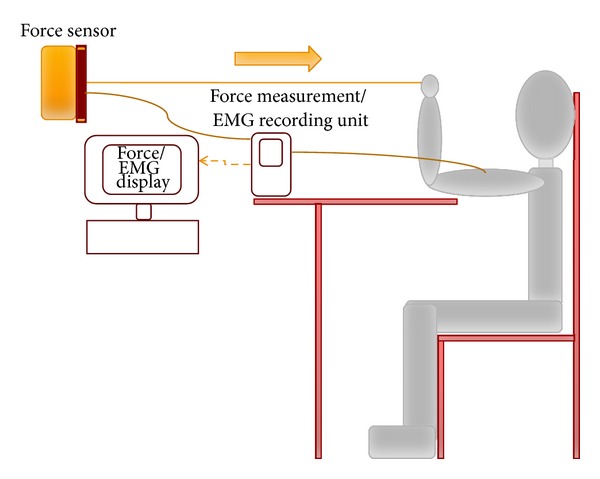
Illustration of the experimental setup.

The experiments were approved by the University Human Ethics Committee. The experiments were performed in accordance with Declaration of Helsinki of 1975, as revised in 2004. During the experiments, the volunteers were seated such that their feet were flat on the floor, the upper arm rested horizontally on an adjustable desk, and the forearm was vertical (refer to Figure 1) with the elbow at 90 degrees. A wall mounted force sensor (*S*-type force sensor—INTERFACE SM25) was attached to a comfortable hand sized ring with a flexible steel wire padded for comfort and the ring was held on the palm of the participant. The output of the force sensor was recorded alongside the sEMG recording on the Delsys acquisition system. Before the start of the experiment, the experimental procedures were explained to the participants and trial runs were performed to familiarize them with the experiment.

*Determining Maximum Voluntary Contraction (MVC)*. To determine the maximal voluntary contraction (MVC), three maximal contractions of 5 seconds were performed with 120 seconds rest time between each effort. The participants pulled on the ring and the force of contraction was recorded. The force output was displayed on the screen and the participants were encouraged to exert their maximal muscle force and steadily maintain the force. The average of the three readings was considered to be the MVC. If there were any outliers, the experiment was repeated. Preliminary experiments were conducted to check the influence of triceps and brachioradialis muscles on biceps during the contraction by recording sEMG of these muscles. If the muscle activity of these muscles was greater than 5% of MVC of biceps (estimated using RMS of sEMG), the participants were reseated and assisted in resting these muscles.

After confirming that the triceps and brachii were silent and the activity was concentrated on the biceps muscle, the experiments were conducted during which the participants performed three sets of isometric contractions at 50%, 75%, and 100% MVC, respectively. Force of contraction was displayed on the computer to give them feedback and assist them in maintaining steady force. Participants were asked to perform the contractions as long as they could and until they experienced fatigue and pain. The trial was terminated when the subject was unable to exert the required force, and the exerted force dropped below 80% of the target or when the subjects experienced pain, whichever occurred earlier. The pain was subjectively measured using a numeric rating pain index scale (PIS) [5, 18], with range from 0 to 10 and with PIS of 0 corresponding to “no pain,” and 10 corresponding to “maximum pain.” Subjects were informed of the PIS before the start of the experiment and were given examples to help them understand the scale. The subjects were requested and reminded to report the pain only in the biceps muscle. A score of 8 and above corresponded to the limit of muscle endurance.

**Figure 2:**
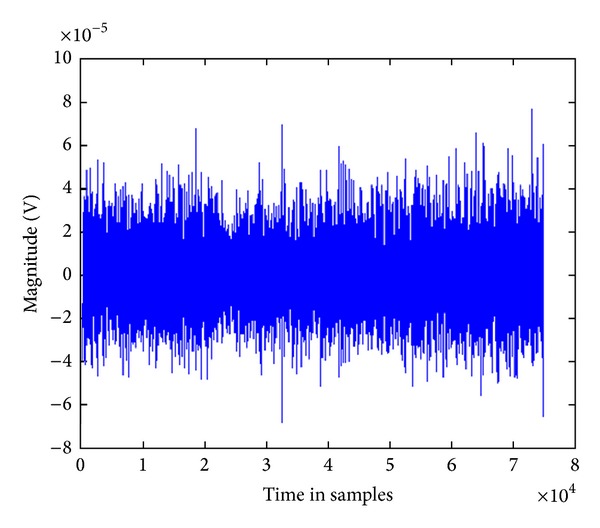
A sample of the recorded raw sEMG signal (time in samples).

The total duration of each contraction was referred to as the endurance period. This was found to be different for different participants and for different levels of muscle contraction. To allow a comparison between different experiments, the time axis was normalized such that the start of the experiment corresponded to *T*_1_ and the end of the exercise was labeled *T*_E_. The participants were given a rest period of 60 minutes between each contraction, but as long as they required. The participants were questioned such that they reported the muscle to be pain-free and rested. The experiments performed in this study were similar to our earlier published protocol [14]. A sample raw EMG signal recorded in the experiment has been shown in Figure 2.

### 2.3. Data Analysis

Data analysis was performed offline on MATLAB 2009a software environment (The MathWorks Inc., Natick, Massachusetts, USA). The first step was the temporal segmentation of the recordings and this was followed by computation of the six features. Regression analysis was performed to determine the linearity of the relationship between each of the features and force of contraction. Finally, *N*-factor analysis of variance (ANOVA) was computed to determine the statistical significance of the relationship.

#### 2.3.1. Temporal Segmentation

All sEMG recordings were divided into 1 second long segments (1000 samples) with overlap of 100 samples using a moving window. The average of the feature of all the windows in the corresponding segment was computed and labeled according to the segment number. The first segment, corresponding to the start of the exercise, was labeled as that of the rested muscle, while the final segment corresponded to the fatigued muscle, at the limit of endurance.

#### 2.3.2. Computation of Features

The following features were computed.

*(i) Normalized Spectral Moments (NSM5)*. The spectral fatigue indices (FI_nsm5_) proposed by Dimitrov et al. [12] and Dimitrova et al. [13] are the measure of the normalised spectral moments as follows:

(1) $$\begin{array}{c} \text{F}{\text{I}}_{\text{nsm}5}=\frac{\int_{f_{1}}^{f_{2}}f^{-1}\cdot \text{PS}\left(f\right)\cdot df}{\int_{f_{1}}^{f_{2}}f^{5}\cdot \text{PS}\left(f\right)\cdot df}, \end{array}$$

where PS(*f*) is the EMG power spectrum, *f*_1_ = 8 Hz, *f*_2_ = 500 Hz, and “·” represents the multiplication factor (see [12, 13] for further details). 

*(ii) Median Frequency (MDF)*. Median frequency is the particular frequency that divides the power spectrum into two sections of equal areas (see [19, 20] for details).

*(iii) Root Mean Square (RMS)*. Root mean square (RMS) is the quadratic mean and a statistical measure of the magnitude of a time varying signal and is computed using the following equation:

(2) $$\begin{array}{c} \text{RMS}=\sqrt{\frac{1}{N}\overset{N}{\underset{i=1}{\sum }}x_{i}^{2}}, \end{array}$$

where *N* is the number of samples in the segment and *x* is the sEMG signal.

*(iv) Normalised RMS (NRMS)*. RMS of sEMG for each participant (at 75% and 50% MVC) was normalized with respect to the RMS of sEMG corresponding to their MVC of the rested muscle and was computed using the following equation:

(3) $$\begin{array}{c} \text{NRMS}=\frac{\text{RM}{\text{S}}_{75\%,\;50\%\;\text{MVC}}}{\text{RM}{\text{S}}_{\text{MVC}}}. \end{array}$$

*(v) Waveform Length (WL)*. Wavelength is the measure of the length of the signal and is computed using the following equation:

(4) $$\begin{array}{c} \text{WL}=\frac{1}{N}\overset{N-1}{\underset{i=1}{\sum }}\left|x_{i+1}-x_{i}\right|, \end{array}$$

where *N* is the number of samples in the segment and *x* is the sEMG signal (in samples).

*(vi) Increase in Synchronization (IIS) Index*.* Increase in synchronization* (IIS) index is the measure of independence between two signals. In this study IIS index was computed using the EMG recordings from the two channels (two sensors) [14] using 1 second window length similar to other features. The computation of IIS index has been explained in detail in our earlier publications [14, 21–23]. The signal was filtered into four narrow subband components of equal bandwidth (band pass filter with 125 Hz frequency band). Independent component analysis (ICA) was performed on each subband component and the resultant *n* = 4 unmixing or separating square matrices, *W*_*n*_. The global matrix, *G*, was estimated as the product of the *n*th unmixing matrix and the inverse of the (*n* + 1)th unmixing matrix. Average of ||*G*|| of all the time windows in the segment was computed to obtain $\bar{||G||}$, and IIS index corresponding to the segment was obtained by computing $\log\bar{||G||}$, the normalized determinant of the global matrix.

#### 2.3.3. Regression Analysis

To determine the relationship between the level of contraction (force) and the features, regression analysis was performed. For this analysis, the features computed from the sEMG were recorded during the initial state of the muscle contraction. Preliminary analysis showed that the relationships were linear in nature. Based on this criterion, the relationship between the level of contraction during* initial state* of maintaining the force and the features was computed using linear regression analysis with 95% confidence intervals.

#### 2.3.4. Statistical Analysis

The statistical significance of the effect and the relationship between the different factors on each of the six features of sEMG was studied. Three-way analysis of variance (ANOVA) with interaction conducted for each of the 6 features with 95% confidence interval (*P* < 0.05) was performed. Kurtosis measures and skew tests were performed to check and confirm the underlying assumptions of ANOVA in analysing the data. The three factors were (i) difference between subjects, (ii) level of contraction (% MVC), and (iii) muscle fatigue. In this study, “localised muscle fatigue” was defined as the condition when the subject was unable to perform the level of contraction and reported a high level of discomfort (>8 on PIS).

The factor,* subject*, was considered as a random factor. ANOVA model was designed to identify the significance of the different factors on each of the six features of sEMG. The result of this analysis would indicate the features that are best able to identify the effect due to the factor and also determine the strength of the relationship between the different factors.

## 3. Results

Table 1 is the summary of the results from the three factor main effects using the ANOVA model, repeated for each of the six features. The three factors considered were (i) subject, (ii) level of contraction (% MVC), and (iii) localised muscle fatigue. The effect of localised muscle fatigue was defined as the difference between near the start and near the end of the exercise. From Table 1, it is observed that the level of contraction (% MVC) as a main effect had significant effect on NSM5 (*P* < 0.01), RMS (*P* < 0.05), NRMS (*P* < 0.05), and IIS index (*P* < 0.05). From this table, it is also observed that the statistical significant relationship of muscle fatigue was only with IIS index of sEMG (*P* < 0.01). The effect on NMRS at 100% MVC was ignored for the statistical and regression analysis because this was the basis for normalisation of the rest of the data.

**Figure 3:**
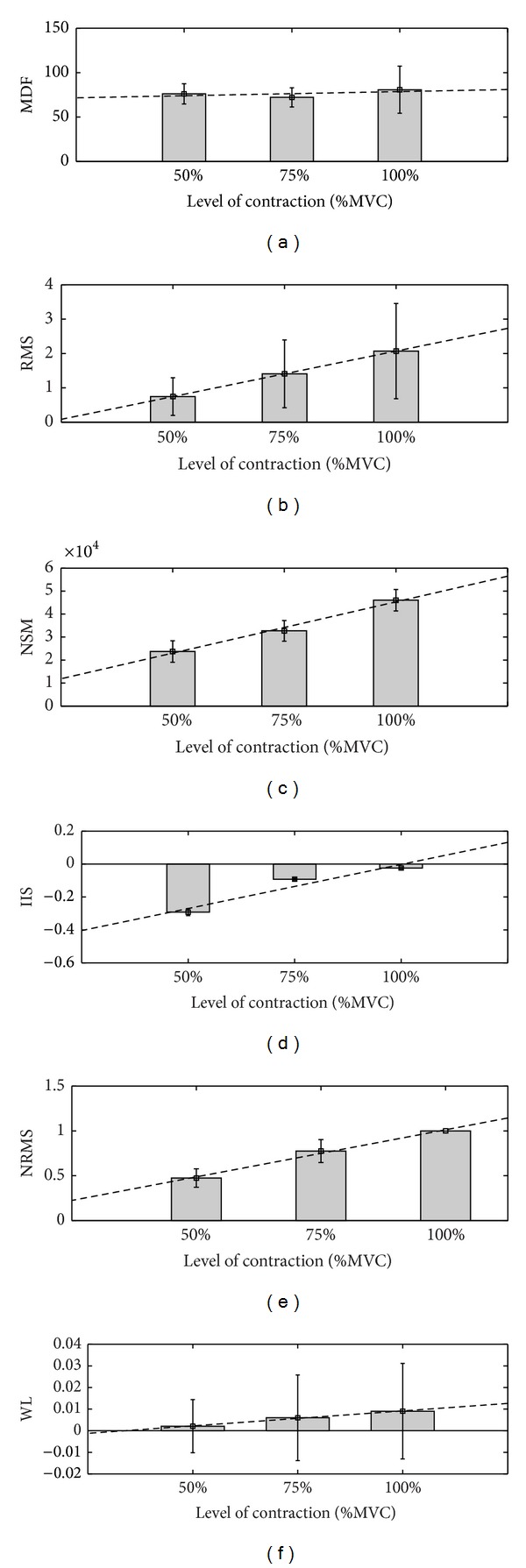
Average (± SD) of six features of sEMG and level of contraction for the rested muscle: (a) MDF, (b) RMS, (c) normalised spectral index (NSM5), (d) IIS index, (e) normalised RMS, and (f) WL.

Table 2 shows the results of the regression analysis performed using the features of sEMG computed from the initial state of the muscle contraction. From this table, it is observed that the relationship of NSM5 of sEMG with muscle force was the most linear in comparison with other features (*R* ^2^ = 0.96). NRMS also showed a good linearity relationship with *R* ^2^ value of 0.93. The bar plots showing the mean values (and standard deviation) of the different features with level of contraction (% of MVC) are shown in Figure 3. These plots confirm the observations from Tables 1 and 2 that NSM5 has a strong linear relationship with force of muscle contraction.

## 4. Discussion and Conclusion

The association between the sEMG and force of muscle contraction is well accepted. Different features of sEMG such as RMS, median frequency, normalized spectral moments, and wavelength [7–9, 11–13] have been studied in relation with the muscle force. However, none of these studies have reported a comparison between the different features to determine the statistical significance of these relationships nor have these analyzed the linearity of these relationships. Such an analysis is important for direct interpretation of sEMG to estimate the force of muscle contraction.

This study has experimentally studied and compared the relationship of muscle force and muscle fatigue with each of the six well-accepted features of sEMG. Analysis of variance (ANOVA) was conducted to determine the effect of three main different factors: level of muscle contraction (% MVC), muscle fatigue, and intersubject variation. ANOVA results show that RMS (*P* < 0.05) and NSM5 (*P* < 0.01) of sEMG were significantly affected by the level of muscle contraction (% MVC). The results also indicate that IIS index (*P* < 0.01) was significant in identifying the muscle endurance limit.

It is observed from the results that the intersubject variation was significant for RMS, NRMS, and WL, while the variation was not significant for NSM5 and IIS index (*P* > 0.05). Features such as RMS, NRMS, and WL are associated with the amplitude of the signal, and the results indicate that there is a significant variation in the amplitude of sEMG between subjects. However, other features that are based on spectrum such as NSM5 or entropy dependent such as IIS index do not have significant differences between subjects.

The linearity analysis indicates that the most linear relationship of force of contraction (ranging from 50 to 100% MVC) was with NSM5 (*R* ^2^ = 0.96), followed by normalized RMS (*R* ^2^ = 0.93). The linearity relationship between RMS of the signal and force of contraction was poor (*R* ^2^ = 0.71). This was also observed from the plots (Figure 2). The results suggest that the relationship between NSM5, a normalized measure of the spectrum of the signal [12], and force of muscle contraction (% MVC) is the most significant and linear. The results also indicate that, for biceps, NSM5 do not require any normalization or calibration. This indicates that NSM5 is suitable to estimate the level of muscle contraction compared with other features [6, 24] even though this has not been reported in literature.

While earlier studies have identified NSM5 to be a fatigue index [12, 13], this study has shown that NSM5 of sEMG is the measure of force of muscle contraction. This may be attributed to the spectrum of sEMG being significantly influenced by the rate of muscle stimulation and thus with force of muscle contraction. This study has also confirmed that IIS index [14] is the most suitable indicator of muscle being at the limit of endurance and is fatigued. The study also found that the advantage of both NSM5 and IIS was that these did not require any normalization.

## Figures and Tables

**Table 1:** Three-way ANOVA for each of the six features with 95% confidence interval. The three factors and their considered interactions are subject, level of contraction (% MVC), and muscle fatigue factor (initial and final segments of the exercise), for isometric contraction and for the six features of sEMG.

Features	Effect of subject *F*-value (*P*)	Effect of level of contraction (% MVC) *F*-value (*P*)	Effect of muscle fatigue *F*-value (*P*)
MDF	1.04 (**0.31**)	0.68 (**0.41**)	1.12 (**0.42**)
NRMS	1.44 (**0.01**)*	7.15 (**0.01**)*	0.51 (**0.48**)
RMS	2.51 (**0.01**)*	5.24 (**0.04**)*	0.68 (**0.56**)
WL	1.05 (**0.02**)*	2.58 (**0.1**)	0.85 (**0.42**)
NSM5	0.94 (**0.548**)	14.22 (**0.001**)^#^	2.86 (**0.093**)
IIS index	0.78 (**0.96**)	4.57 (**0.031**)*	4288 (**0.001**)^#^

*Significant *P* < 0.05; ^#^ *P* < 0.01.

**Table 2:** Mean values *R* ^2^ performed using regression analysis (linear: with force as the factor)—95% confidence interval.

Features	*R* ^2^	*P*
NRMS	0.93 (±0.05)	0.02
RMS	0.71 (±0.12)	0.054
MDF	0.32 (±0.14)	0.45
WL	0.51 (±0.11)	0.1
NSM5	0.96 (±0.04)	0.001^#^
IIS	0.79 (±0.09)	0.04

^#^ *P* < 0.01.
